# Analysing breast cancer survivors’ acceptance profiles for using an electronic pillbox connected to a smartphone application using Seintinelles, a French community-based research tool

**DOI:** 10.3389/fphar.2022.889695

**Published:** 2022-09-27

**Authors:** Catherine Goetzinger, Caroline Alleaume, Anna Schritz, Bernard Vrijens, Marie Préau, Guy Fagherazzi, Laetitia Huiart

**Affiliations:** ^1^ Deep Digital Phenotyping Research Unit, Department of Precision Health, Luxembourg Institute of Health, Strassen, Luxembourg; ^2^ University of Luxembourg, Faculty of Science, Technology and Medicine, Esch-sur-Alzette, Luxembourg; ^3^ Santé Publique France, Paris, France; ^4^ Competence Center for Methodology and Statistics, Luxembourg Institute of Health, Strassen, Luxembourg; ^5^ AARDEX Group & Department of Public Health, Liège University, Liège, Belgium; ^6^ Institut de Psychologie, Université Lumière Lyon 2, Lyon, France

**Keywords:** medication adherence, medication adherence enhancing interventions, eHealth, breast cancer, user-centered design, patient adherence

## Abstract

**Introduction:** Up to 50% of breast cancer (BC) survivors discontinue their adjuvant endocrine therapy (AET) before the recommended 5 years, raising the issue of medication non-adherence. eHealth technologies have the potential to support patients to enhance their medication adherence and may offer an effective way to complement the healthcare. In order for eHealth technologies to be successfully implemented into the healthcare system, end-users need to be willing and accepting to use these eHealth technologies.

**Aim:** This study aims to evaluate the current usability of eHealth technologiesin and to identify differences in BC SURVIVORS BC survivors accepting a medication adherence enhancing eHealth technology to support their AET to BC survivors that do not accept such a medication adherence enhancing eHealth technology.

**Methods:** This study was conducted in 2020 including volunteering BC survivors belonging to the Seintinelles Association. Eligible participants were women, diagnosed with BC within the last 10 years, and been exposed to, an AET. Univariable and multivariable logistic regression analyses were performed to investigate medication adherence enhancing eHealth technology acceptance profiles among BC survivors. The dependent variable was defined as acceptance of an electronic pillbox connected to a smartphone application (hereafter: medication adherence enhancing eHealth technology).

**Results:** Overall, 23% of the participants already use a connected device or health application on a regular basis. The mean age of the participants was 52.7 (SD 10.4) years. In total, 67% of 1268 BC survivors who participated in the survey declared that they would accept a medication adherence enhancing eHealth technology to improve their AET. BC survivors accepting a medication adherence enhancing eHealth technology for their AET, are younger (OR = 0.97, 95% CI [0.95; 0.98]), do take medication for other diseases (OR = 0.31, 95% CI [0.13; 0.68]), already use a medication adherence enhancing eHealth technology or technique (OR = 1.74, 95% CI [1.06; 2.94]) and are willing to possess or currently possess one or more connected devices or health applications (OR = 2.89, 95% CI [2.01; 4.19]).

**Conclusion:** Understanding acceptance profiles of BC survivors is fundamental for conceiving an effective eHealth technology enhancing AET among BC survivors. Hence, such profiling will foster the development of personalized medication adherence enhancing eHealth technology.

## 1 Introduction

Breast cancer (BC) is the most common cancer among women, as 355,000 are estimated to be diagnosed with BC each year in Europe (International Agency for Research on Cancer et al., 2020). The majority (80%) of BC patients are hormone receptor–positive and most (>90%) have stage I to III and are eligible for adjuvant endocrine therapy (AET) (Partridge et al., 2003).

The shift, that BC survivors experience from the acute phase of treatment (e.g., surgery, chemotherapy, radiotherapy) to the post-acute phase (e.g., AET), is associated with social and medical challenges (Kantsiper et al., 2009; Hurtado-de-Mendoza et al., 2017; Goetzinger et al., 2020). Patients recurrently reported the need for increased support in terms of AET management (adherence and side effects) as well as increased patient–healthcare provider communication and follow-up (Finitsis et al., 2019; Pouls et al., 2021). During this post-acute treatment period, most BC survivors report anxiety, fear, and struggle to find their way back into everyday life. In addition, BC survivors usually do not visit their oncologist for a relatively long period during the post-acute treatment phase (Ringwald et al., 2017; Goetzinger et al., 2020). Thus the value of HCP support during this survivorship period of BC patients is undebatable for medication adherence and disease management (Kini and Michael Ho, 2018).

Medication adherence is a dynamic behaviour influenced by various factors (Sabaté and World Health Organization, 2003; Kardas et al., 2013) and is defined as the process by which patients take their medication as prescribed. This medication adherence process is further categorized into three distinct phases: 1. *Initiation* (patient takes the first dose of prescribed medication), 2. *Implementation* (the extent to which a patient’s actual dosing corresponds to the prescribed dosing regimen, from initiation until the last dose is taken) and 3. *Discontinuation* (occurs when the patient stops taking the prescribed medication, for whatever reason(s)) (Vrijens et al., 2012). Previous work demonstrated that 30%–50% of BC survivors discontinue their AET before the recommended 5 years end depending on the AET agent and method of medication adherence measurement (Huiart et al., 2011). Moreover, it was shown that AET reduces BC recurrence rate by 50% and mortality by a third (Davies et al., 2011; Pistilli et al., 2020). Therefore, it is key to identify AET non-adherence, to reduce the risk for poorer health outcomes (Pistilli et al., 2020). To date, there is no gold standard to identify non-adherence. Indirect methods such as pharmacy prescription refills or patient-administered questionnaires are mostly used, yet fail to measure the real medication intake or even overestimate adherence (Lu et al., 2018).

The World Health Organization defines eHealth ‘as the cost-effective and secure use of information and communications technologies in support of health and health-related fields, including health-care services, health surveillance, health literature, and health education, knowledge and research (World Health Organization, 2022)’. Concerning the field of medication adherence research and eHealth, medication adherence technologies (MATech) such as electronic pillboxes or smartphone applications have been developed (Ahmed et al., 2018). Car et al. highlighted that these MATechs are the future for self-management of treatment and medication adherence monitoring (Car et al., 2017). A systematic review by Nieuwlaat et al. showed that MATechs are most effective if multiple components, trying to overcome barriers to adherence by means of tailored ongoing support from allied health professionals are used (Nieuwlaat et al., 2014). Nevertheless, the most effective interventions did not lead to large improvements in adherence or clinical outcomes (Hadji et al., 2013; Finitsis et al., 2019; Rosenberg et al., 2020). This is because most of those interventions were created without the involvement of the end-user, whereas patient involvement is key in research and implantation into the healthcare setting (De Geest et al., 2020; Aguayo et al., 2021). Thus, BC survivor involvement is key to conceive effective MATechs to enhance AET. In order to personalize medication adherence enhancing interventions for subtypes of BC survivor users, it is important to profile the acceptance of BC survivors to use medication adherence enhancing eHealth technology for AET enhancement.

Therefore, the present study aims to 1) evaluate the current usability of eHealth technologies in BC survivors and to 2) identify differences in BC survivors accepting medication adherence enhancing eHealth technology to enhance their AET to BC survivors that do not accept such a medication adherence enhancing eHealth technology. In this study, we define medication adherence enhancing eHealth technology as an electronic pillbox connected to a smartphone application.

## 2 Method

### 2.1 Study design

A cross-sectional, e-survey was conducted from July to December 2020 among BC survivors from the French Seintinelles platform (www.seintinelles.com). Seintinelles is a non-profit community-based research platform, developed in collaboration with psycho-oncologists to facilitate the implication of patients into cancer research (Bauquier et al., 2017; Pannard et al., 2020). Volunteering citizens, regardless of their current health condition and/or cancer type, can participate in this platform, comprised of over 8000 BC patients (in 2020), the target population of the present study. Thus, this platform has the ability to recruit a large number of participants in a very limited time.

### 2.2 Recruitment and study population

Seintinelles sent an email to all its BC members, informing them about the study objectives, along with the information sheet (Supplementary Appendix 1). If they were interested in participating, they were asked to complete a short questionnaire on the website to verify that they met all the inclusion criteria (Supplementary Appendix 2). Inclusion criteria for this e-survey were:

- Women,
- BC diagnosed within the last 10 years,
- at least temporarily exposed to an AET.

If participants met all inclusion criteria and still wanted to participate, they signed an e-consent form before starting the e-survey (Supplementary Appendix 2).

### 2.3 e-survey

The e-survey used within the present study aims to establish a state of art on current eHealth usability and potential acceptability of medication adherence enhancing eHealth technology in BC survivors.

The e-survey consists of about 30 questions and required participants’ attention for at least 20 min. They had the option to interrupt the questionnaire, and could save their answers to continue later. There were no incentives given to participants. BC survivors (N = 2) proofread the final version of the e-survey. CG and CA as well as employees of Seintinelles pre-tested the e-survey with respect to technical errors and incorrect utilisation of question filters. While conducting the e-survey, participants could only see one question at a time. It was mandatory to answer the question in order to get to the next. This method was used to ensure that no questions was left unanswered.

### 2.4 Measurement

The e-survey was subdivided into five sections to collect data on socio-demographic characteristics, health status and disease experience, medication adherence, eHealth utilization and a specific section on medication adherence enhancing eHealth technology. For more information, Supplementary Appendix 3 illustrates the structure and definitions of the e-survey.

#### 2.4.1 Sociodemographic characteristics

The first section of the e-survey collected data on participants’ age, marital status, having children and number of children. In addition, participants responded to questions asking about their educational, professional and financial status. These items were adapted from the questionnaire used in Vican 5, a French nationwide population-based questionnaire aiming to explore life 5 years after cancer diagnosis (Bauquier et al., 2017).

#### 2.4.2 Health status and disease experience

The second section investigated participant’s general health status and their experience with BC in the acute phase of treatment. These questions were either developed by CA and CG or taken from Vican 5 (Bouhnik et al., 2015).

#### 2.4.3 AET adherence

The third section analyzed the adherence to AET in terms of persistence and if discontinuation for which reasons. In addition, this third section investigated experienced side effects and use of support by psychologists or alternative medicine. Furthermore, current techniques or eHealth technologies used to support participants with their AET intake were investigated.

This section sums up by evaluating the patient–physician relationship and communication. CA and CG developed these questions.

#### 2.4.4 eHealth utilization

Section 4 evaluated current eHealth utilisation. This section of the questionnaire-survey was based on a self-administered qualitative questionnaire used in social psychology science in the DISCO trial (DISpositif COnnecté’, connected device in English) investigating the use and acceptability of connected devices in breast cancer (Touillaud et al., 2021). As in the questionnaire from the DISCO trial, we provided the participant with two definitions, explaining ‘connected device’ and ‘mobile application’. In contrast to the DISCO trial questionnaire, the present study focuses more precisely on adherence to OHT in BC survivors, thus additional items, created by CG and CA, were based on the results found by Goetzinger et al. (2020).

#### 2.4.5 Medication adherence enhancing eHealth technology

The fifth section investigated acceptability and related barriers and facilitators to acceptability and usability of a proposed medication adherence enhancing eHealth technology supporting AET management in BC survivors. This paper will only focus on the first question of this section, as it is the dependent variable used for the univariable and multivariable logistic regression analyses.

### 2.5 Dependent variable

The dependent variable ‘Acceptance of a Medication adherence enhancing eHealth technology (electronic pillbox connected to a smartphone application)’’ (1 = yes, 0 = no) was computed from ‘Would you accept to use an electronic blister connected to an application on your phone to support your AET treatment’. Hence, we categorized the following answers together to receive a binary variable;

‘Yes’ includes the following answer options:

• ‘Yes, I accept voluntarily’,
• ‘Yes, if my Doctor asks me to’,
• ‘Yes, depending on the information provided’.

‘No’ includes these answer options;

• ‘No, I do not trust connected devices’,
• ‘No, I don’t know how to use new technology’,
• ‘No, I don’t have a smartphone and I don’t want one’,
• ‘No, for other reasons’.

### 2.6 Ethical provision

The study received approval by the National Commission for Information and Freedoms (Commission nationale de l’informatique et des libertés, CNIL: 1955704) and the Sud-EST II data protection committee (Comité de Protection des données, Numéro EudraCT: 2020-A00665-34).

### 2.7 Statistical analysis

This study uses descriptive statistics to characterize the study population and to highlight current patterns of eHealth use in BC survivors. Univariable and multivariable logistic regression analyses were performed to evaluate differences in BC survivors that accept an electronic blister connected to app to support AET adherence with those that do not. Odds ratios were used as the measure of association to compare the strength of the correlation between ‘Medication adherence enhancing eHealth technology acceptance’ and relative predictors. We performed a both-way stepwise logistic regression analysis to investigate factors that are significantly associated with accepting an electronic blister connected to the app to support AET adherence. The final model was retained as the lowest AIC was achieved. Significance was accepted at a *p*-value lower than 0.05, with a 95% Confidence Interval. We used the R software version 4.0.3 including the ‘ISwR’, ‘oddsratio’, ‘StepReg’, ‘forestplot’ and ‘dyplr’ packages to analyse the data and conceive the figure. This study used only completed questionnaires in order to avoid weighing and computation of missing values.

## 3 Results

Overall, 1,516 eligible Seintinelles members started the questionnaire, 1268 BC survivors responded to the complete online questionnaire and were used for the analysis. No missing values were recorded in our dataset as participants could only proceed in the questionnaire when the previous question was answered.

The overall study sample is on average 52.7 years (SD 10.4) old, over half is married (73.9%), and employed (60.3%) (Table 1). Furthermore, 46% of the overall sample reported good general health, and more than half of the study sample did not use any other medication for other diseases (52.8%). 21% of the participants were diagnosed with BC before 2012, 12% in 2015 and 21% after 2018. About a third (32.6%) of the BC survivors state that their BC does have ‘some effect’ on their life. Only 7.7% of the BC survivors evaluate themselves to be able to control their disease and almost 40% claim to have very good knowledge about the disease. Moreover, 88% highlighted that they had no BC recurrence up to the date of the questionnaire completion.

**TABLE 1:** Descriptive characteristics of BCS (Seintinelles study, 2020).

	Overall (N = 1,268)	Acceptance of an electronic blister connected to an app		
		Yes (N = 845)	No (N = 423)	*p-value*
Total	100%	66.6%	33.4%	
**Sociodemographic characteristics**				
Age (mean, SD)	52.7 +-10.4	51.4 +- 10.3	55.3 +- 10.3	<0.001
Marital status				
Single	156 (12.3%)	95 (11.2%)	61 (14.4%)	0.031
Married	937 (73.9%)	646 (76.4%)	291 (68.8%)	
Widow	34 (2.7%)	19 (2.3%)	15 (3.6%)	
Divorced	141 (11.1%)	85 (10.1%)	56 (13.2%)	
Children				
Yes	1,021 (80.5%)	686 (81.2%)	335 (79.2%)	0.443
			
No	247 (19.5%)	159 (18.8%)	88 (20.8%)	
Education				
High school degree	205 (16.2%)	128 (15.2%)	77 (18.2%)	0.144
Bachelor or equivalent	390 (30.8%)	268 (31.7%)	122 (28.8%)	
Master or equivalent	554 (43.7%)	371 (43.9%)	183 (43.3%)	
Professional diploma	94 (7.4%)	66 (7.8%)	28 (6.6%)	
Other	25 (1.9%)	12 (1.4%)	13 (3.1%)	
Professional status				
Employed	764 (60.3%)	538 (63.7%)	226 (53.4%)	<0.001
Sick leave	61 (4.8%)	36 (4.3%)	25 (5.9%)	
Job hunting	49 (3.7%)	31 (3.7%)	18 (4.3%)	
Retired	248 (19.6%)	138 (16.3%)	110 (26.0%)	
Self-employed	78 (6.2%)	51 (6.0%)	27 (6.4%)	
Other	68 (5.4%)	51 (6.0%)	17 (4.0%)	
Financial status				
At ease	948 (74.8%)	627 (74.2%)	321 (75.9%)	0.560
Difficult	320 (25.2%)	218 (25.8%)	102 (24.1%)	
**Health status and experience with breast cancer**				
General health status				
Very good	164 (12.9%)	108 (12.8%)	56 (13.2%)	0.379
Good	586 (46.2%)	403 (47.7%)	183 (43.3%)	
Ok	462 (36.4%)	295 (34.9%)	167 (39.5%)	
Bad	56 (4.5%)	39 (4.6%)	17 (4.0%)	
Medication for other disease				
Daily	456 (35.9%)	294 (34.8%)	162 (38.3%)	<0.001
Regularly	39 (3.1%)	15 (1.8%)	24 (5.6%)	
In case of need	104 (8.2%)	69 (8.2%)	35 (8.3%)	
No	669 (52.8%)	467 (55.2%)	202 (47.8%)	
Year of diagnosis				
<2012	261 (20.6%)	164 (19.4%)	97 (22.9%)	0.113
2013	119 (9.4%)	76 (9.0%)	43 (10.2%)	
2014	144 (11.4%)	93 (11.0%)	51 (12.1%)	
2015	153 (12.1%)	95 (11.2%)	58 (13.7%)	
2016	154 (12.1%)	101 (12.0%)	53 (12.5%)	
2017	169 (13.3%)	121 (14.3%)	48 (11.3%)	
>2018	268 (21.1%)	195 (23.1%)	73 (17.3%)	
Quality of life/BC impact on life (bc->BC)				
No effect at all	163 (12.9%)	97 (11.5%)	66 (15.6%)	0.027
Does not affect much	363 (28.6%)	231 (27.3%)	132 (31.2%)	
Some effect	414 (32.6%)	283 (33.5%)	131 (30.9%)	
Does effect	245 (19.3%)	180 (21.3%)	65 (15.4%)	
Does effect severely	83 (6.6%)	54 (6.4%)	29 (6.9%)	
Control over BC				
No control	194 (15.3%)	115 (13.6%)	79 (18.7%)	0.027
Not very much control	302 (23.8%)	217 (25.7%)	85 (20.1%)	
Some control	414 (32.6%)	289 (34.2%)	125 (29.5%)	
Control	260 (20.5%)	165 (19.5%)	95 (22.5%)	
A lot of control	98 (7.7%)	59 (7.0%)	39 (9.2%)	
Knowledge of BC				
No knowledge	33 (2.6%)	20 (2.4%)	13 (3%)	0.262
No real knowledge	65 (5.1%)	42 (4.9%)	23 (5%)	
Some knowledge	270 (21.3%)	190 (22.5%)	80 (19%)	
Good knowledge	412 (32.5%)	283 (33.5%)	129 (300%)	
Very good knowledge	488 (38.5%)	319 (36.7%)	178 (42%)	
BC recurrence				
Yes	149 (11.8%)	102 (12.1%)	47 (11.1%)	0.683
No	1,119 (88.2%)	743 (87.9%)	376 (88.9%)	
**Treatment adherence**				
Taking an AET				
Yes	882 (69.6%)	604 (71.5%)	278 (65.7%)	0.042
No	386 (30.4%)	241 (28.5%)	145 (34.3%)	
Side-effects				
Yes	1,160 (91.5%)	776 (91.8%)	384 (90.8%)	0.598
No	108 (8.5%)	69 (8.2%)	39 (9.2%)	
AET interruptions				
Yes	117 (9.2%)	71 (8.4%)	46 (10.9%)	0.183
No	1,151 (90.8%)	774 (91.6%)	377 (89.1%)	
**Patient-Physician communication**				
GP implication in bc follow-up				
Yes, regularly	383 (30.2%)	261 (30.9%)	122 (28.8%)	0.197
Yes, occasionally	287 (22.6%)	202 (23.9%)	85 (20.1%)	
Yes, exceptionally	239 (18.9%)	149 (17.6%)	90 (21.3%)	
No, never	359 (28.3%)	233 (27.6%)	126 (29.8%)	
Bcs′ satisfaction on physicians information given regarding the: nature of the treatment				
Very unsatisfying	87 (6.9%)	51 (6.0%)	36 (8.5%)	0.067
Unsatisfying	196 (15.5%)	132 (15.6%)	64 (15.1%)	
Correct	433 (34.1%)	273 (32.3%)	160 (37.8%)	
Satisfying	353 (27.8%)	250 (29.6%)	103 (24.4%)	
Very satisfying	199 (15.7%)	139 (16.5%)	60 (14.2%)	
Expected benefits of the treatment				
Very unsatisfying	58 (4.6%)	34 (4.0%)	24 (5.7%)	0.017
Unsatisfying	143 (11.3%)	93 (11.0%)	50 (11.8%)	
Correct	405 (31.9%)	249 (29.5%)	156 (36.9%)	
Satisfying	429 (33.8%)	306 (36.2%)	123 (29.1%)	
Very satisfying	233 (18.4%)	163 (19.3%)	70 (16.5%)	
Treatment side-effects				
Very unsatisfying	198 (15.6%)	125 (14.8%)	73 (17.3%)	0.077
Unsatisfying	342 (27.0%)	227 (26.9%)	115 (27.2%)	
Correct	364 (28.7%)	231 (27.3%)	133 (31.4%)	
Satisfying	247 (19.5%)	182 (21.5%)	65 (15.4%)	
Very satisfying	117 (9.2%)	80 (9.5%)	37 (8.7%)	

At the time of the questionnaire, 69.6% of the BC survivors were taking an AET, 91.5% experienced side effects and 9.2% interrupted their AET. Most women stated that their GP is somewhat implicated in their BC follow-up. A third (33.8) of the BC survivors stated that the information provided by their physician regarding the benefits of their AET is satisfying.

### 3.1 Current eHealth use among BC survivors

Approximately 38% of the included BC survivors did already possess one or more connected devices or health applications and 39% of those use these tools every day (Table 2). 18.7% of these women use these tools to motivate themselves, followed by 14.3% to monitor their health. Current techniques or devices to help BC survivors to adhere to their AET are specific locations to store their AET blister (47.2%), phone alarm (13.0%) and Pillbox (13.3%). About 12% of the BC survivors use at least two of those aids regularly. Most participants (90.3%) claim that these aids help them to adhere to their AET.

**TABLE 2:** Current eHealth use of BCS and acceptance to use a connected electronic blister with an app to manage AET (Seintinelles study, 2020).

	Overall (N = 1,268, %)
**Do you possess 1 or more connected devices or health applications?**	
No, it doesn’t interest me	603 (47.6%)
No, but I know someone close to me who uses them and I am interested	105 (8.3%)
No, but I plan to get one within the next 6 months	76 (6.0%)
Yes but I do not use them	102 (8.0%)
Yes I use them for 1 year	92 (7.2%)
Yes I use them already longer than a year	290 (22.9%)
**If yes, how often did you use the connected device or health app in the last 3 months? (N = 382)**	
Never	24 (6.3%)
Less than once a month	52 (13.6%)
1–3 x a month	51 (13.4%)
Once a week	27 (7.1%)
Twice a week	16 (4.2%)
3x a week	20 (5.2%)
More than 3x a week	43 (11.2%)
Everyday	149 (39.0%)
**If used at “least less than once a month” or more, how do these tools help you? (N = 358)**	
To manage my health	19 (5.3%)
To motivate me	67 (18.7%)
To monitor my health	51 (14.3%)
To motivate me and monitor my health	20 (5.6%)
Other reason(s)	52 (14.5%)
No reason	149 (41.6%)
**During your AET, do you use any devices or specific techniques to help you with your treatment? (multiple answers possible)**	
Phone alarm (yes, %)	165 (13.0%)
Pillbox (yes, %)	168 (13.3%)
A specific location to store the blister (yes, %)	599 (47.2%)
The implication of closed one (yes, %)	73 (5.8%)
Application (yes, %)	15 (1.2%)
Other (yes, %)	59 (4.7%)
None (yes, %)	452 (35.7%)
**Nr of medication adherence support devices/specific techniques used**	
0	452 (35.7%)
1	607 (47.9%)
2	153 (12.1%)
>3	56 (4.3%)
**If at least 1-support devices/specific techniques used, do these tools help you to adhere to your medication? (N = 816)**	
Yes	737 (90.3%)
No	30 (3.7%)
I don’t know	49 (6.0%)
**Which of the following features/facts are important for you regarding your medication adherence? (Multiple answers possible)**	
Auto Surveillance (yes)	459 (36.2%)
Information disposition (yes)	554 (43.7%)
Real-time side effect declaration (yes)	630 (49.7%)
Real-time follow-up by health care professional (yes)	499 (39.4%)
Patient-Physician communication (dematerialised) (yes)	522 (41.2%)
Pharmacy Refill Alarm (yes)	304 (24.0%)
Reduce face-to-face consultations (yes)	298 (23.5%)
Personalized follow-up (yes)	518 (40.9%)
Adherence management (yes)	213 (16.8%)
Exchange with others on treatment (yes)	344 (27.1%)
None (yes)	164 (12.9%)
**Would you accept an electronic pillbox connected to an app on your phone to follow your AET (Dependent variable)?**	
Yes, voluntarily	344 (27.1%)
Yes, if asked by my Doctor	109 (8.6%)
Yes, depending on the information I receive	392 (30.9%)
No, I have no confidence in connected health devices	59 (4.7%)
No, I do not know how to use new technologies	17 (1.3%)
No, because I don’t want a smartphone	28 (2.2%)
No, for other reasons	319 (25.2%)

### 3.2 Medication adherence support tool acceptance

Specific features that support medication adherence and are important for BC survivors to use real-time side effect declaration (49.7%), information disposition (43.7%) and dematerialised patient-physician communication (41.2%) among others. Finally, the study showed that 27.1% of the participants would voluntarily accept to use an electronic pillbox connected to an app on their phone to manage their AET.

### 3.3 Factors associated with BC survivors acceptance of an eHealth tool to manage AET

Table 3 illustrates the univariable logistic regression analysis, which analysed factors associated with accepting an electronic pillbox connected to an app to enhance AET among BC survivors. Some of the factors associated with accepting an electronic pillbox connected to an app were age (OR = 0.96, 95% CI 0.95, 0.98), being married (OR = 1.43, 95% CI 1.00, 2.02), retired (OR = 0.53, 95% CI 0.39, 0.71), taking regular medication for other diseases (OR = 0.34, 95% CI 0.17, 0.67) and using more than one support tool for AET adherence (OR = 1.53, 95%CI 0.18, 0.67).

**TABLE 3:** Factors associated with accepting an eHealth tool to manage OHT in BCS (Seintinelles study, 2020).

	Acceptance of an electronic blister connected to an app		
Univariable logistic regression analysis	OR	95% CI	*p*-value
Age (years)	0.96	0.95–0.98	<0.001
Marital Status			
Single	Ref		
Married	1.43	1.00–2.02	0.047
Widow	0.81	0.39–1.74	0.589
Divorced	0.98	0.61–1.56	0.914
Professional Status			
Employed	Ref		
Sick leave	0.61	0.36–1.04	0.065
Job hunting	0.72	0.40–1.34	0.291
Retired	0.53	0.39–0.71	<0.001
Self-employed	0.79	0.49–1.31	0.816
Other	1.26	0.73–2.29	0.427
Medication for other diseases			
Daily	Ref		
Regularly	0.34	0.17–0.67	0.002
In case of need	1.09	0.70–1.72	0.718
No	1.27	0.99–1.64	0.061
Quality of life			
No effect at all	Ref		
Does not affect much	1.19	0.81–1.74	0.367
Some affect	1.47	1.01–2.14	0.044
Does affect	1.88	1.24–2.88	0.003
Does affect severely	1.27	0.74–2.21	0.398
Control over breast BC			
No control at all	Ref	Ref	
Not very much control	1.75	1.20–2.57	0.004
Some control	1.59	1.11–2.27	0.011
Control	1.19	0.81–1.75	0.365
A lot of control	1.04	0.63–1.71	0.879
taking an adjuvant endocrine therapy			
Yes	Ref		
No	0.77	0.60–0.98	0.036
Number of medication adherence support devices/specific techniques used			
1	Ref	Ref	
2	2.09	1.36–3.28	0.001
>3	2.00	1.05–4.14	0.047
0	0.71	0.55–0.92	0.008
BCS′ Satisfaction On Physicians Information Given regarding the			
Nature Of The Treatment	Ref	Ref	
Very unsatisfying	1.46	0.86–2.45	0.157
Unsatisfying	1.20	0.75–1.92	0.437
Correct	1.71	1.05–2.78	0.029
Satisfying	1.64	0.97–2.76	0.065
Expected Benefits Of The Treatment			
Very unsatisfying	Ref	Ref	
Unsatisfying	1.31	0.70–2.45	0.394
Correct	1.13	0.64–1.96	0.676
Satisfying	1,76	0.99–3.07	0.050
Very satisfying	1.64	0.90–2.97	0.100
Treatment Side-Effects			
Very unsatisfying	Ref	Ref	
Unsatisfying	1.15	0.80–1.66	0.446
Correct	1.01	0.71–1.45	0.938
Satisfying	1.64	1.09–2.46	0.007
Very satisfying	1.26	0.78–2.06	0.346
Possession of connected devices or health applications			
No, it doesn’t interest me	Ref	Ref	
No, but I know someone close to me who uses them and I am interested	1.37	0.87–1.92	<0.001
No, but I plan to get one within the next 6 months	0.70	0.20–1.23	0.008
Yes but I do not use them	1.40	0.89–1.97	<0.001
Yes I use them for 1 year	1.35	0.82–1.93	<0.001
Yes I use them already longer than a year	1.11	0.79–1.43	<0.001

**FIGURE 1:**
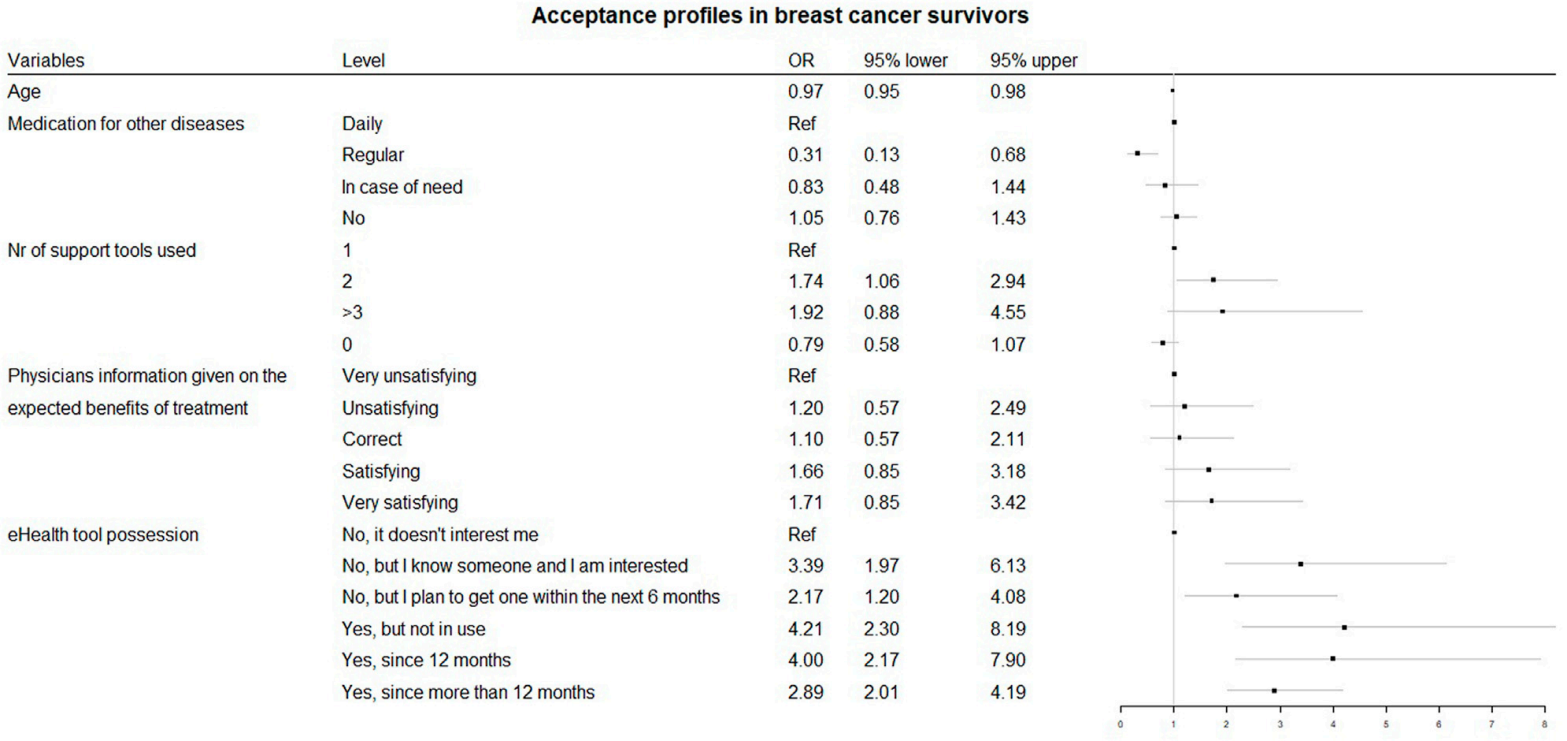
Acceptance profiles in survivors.

Figure 1 highlights the stepwise multivariable logistic regression, presenting factors that are significantly associated with accepting an electronic pillbox connected to an app to enhance AET among BC survivors. The final adjusted model includes ‘Age’, ‘Medication intake for other diseases’, ‘Number of medication adherence support devices used’, ‘BC survivors satisfaction on physicians information given on expected benefits of the treatment’ and ‘Possession of connected devices or health applications’. We performed both forward and backward stepwise regression and both methods selected the same variables.

Hence, accepting an electronic pillbox connected to an app to enhance AET among BC survivors is inversely associated with age (OR = 0.97, 95% CI 0.95, 0.98) and the use of regular intake of other medication compared to no other medication intake (OR = 0.31, 95% CI 0.13, 0.68) (Figure 1). Using at least two medication adherence support tools increases the odds of accepting an electronic pillbox connected to an app to enhance AET among BC survivors (OR = 1.74, 95% CI 1.06, 2.94). Finally, BC survivors using connected devices for more than a year is 2.89 times (95% CI 2.01, 4.19) more likely to accept an eHealth tool to enhance AET compared to those that do not possess or are not interested in connected devices or health applications.

## 4 Discussion

This study investigated differences in BC survivors that accept an electronic pillbox connected to an app to enhance AET with those who do not.

Drewes et al. analysed the correlation between sociodemographic factors, the health status of BC patients and the willingness to use the Internet and apps (Drewes et al., 2016). They found that decisive factors influencing BC patients’ willingness to use new communication technologies are younger, have a large number of people per household, and a short time since breast cancer diagnosis. Other commonly reported barriers to medication adherence across diseases, patient beliefs/perceptions, comorbidities and poor patient–provider communication among others (Konstantinou et al., 2020). We found similar results and add to the current knowledge that polypharmacy positively effects acceptance of a medication adherence enhancing eHealth technology. Furthermore, we found that those patients that have already created an AET adherence habit/technique or are willing to use a smartphone or health applications are more likely to use an AET enhancing eHealth tool. Similar eHealth acceptance trends can be found for patients with cardiometabolic diseases, mental health disorders, infectious diseases (Talal et al., 2019; AshaRani et al., 2021; Gire et al., 2021).

In our study, we found that at the time of the survey, only 1.2% actively used an app yet 67% of the BC survivors would accept to use the proposed electronic pillbox connected to an app to enhance their AET. As Car et al. mentioned, eHealth is the future of medications management in terms of personalisation, monitoring and adherence (Car et al., 2017). To date, digitally delivered interventions including components such as medication and condition education, motivational interviewing, reinforcement and motivational messages led to improvements in medication adherence (Hadji et al., 2013; Nieuwlaat et al., 2014; Finitsis et al., 2019; Rosenberg et al., 2020; Pouls et al., 2021). In addition, qualitative papers showed that patients are ready and willing to integrate eHealth technologies into their daily life to monitor and enhance their health status and medication intake (Currie et al., 2015; Goetzinger et al., 2020). Yet, the challenge we face is to conceive effective eHealth intervention for end-users and implement them into the healthcare sector (Car et al., 2012). Thus integrating patients into the development phase of these eHealth technologies is key to creating feasible tools for the end-user that are implementable into the healthcare setting (Ross et al., 2016; Bauquier et al., 2017; Pannard et al., 2020; Aguayo et al., 2021).

Understanding the disease and/or patient profiles will allow personalising healthcare in the future. Characterising patient groups will allow defining new strategies for individual patients benefiting their needs to optimise health outcomes. Recent research, using profiling principles, found that healthcare for patients with cardiometabolic disease could benefit from more targeted and tailored strategies for the prevention of cardiometabolic diseases at a population level (Fagherazzi et al., 2021). Eventually, post-acute treatment for BC survivors using a medication adherence enhancing eHealth technology can move from a “one-size-fits-all” vision to a tailored follow-up strategy, personalizing care to each BC survivor.

This study evaluated the association between BC survivors characteristics and the acceptance of an eHealth intervention among BC survivors. Hence, the results produced will be fundamental when conceiving an eHealth support tool to enhance AET among BC survivors. Using patient acceptance profiling strategies will allow them to provide them with personalised care and develop effective, sustainable, and implementable eHealth support tools. Future studies should have a closer look into the specific features of such an AET support tool, examine the acceptable time point(s) of intervention and evaluate the implication of HCP. In addition, implementation strategies to adopt these eHealth technologies into the healthcare system need to be investigated.

### 4.1 Limitations

The present study entails several limitations. Also, the present study deals with selection bias, as the Seintinelles platform only includes volunteering members. Meaning the participants showed interest in the study topic, also we observed a high educational level among the study sample. The present study thus provides only a snapshot of characteristics for accepting eHealth tools. Some categories have a small sample and should be regarded with caution.

## 5 Conclusion

This study found that although 1.2% currently used and health related app over two thirds would accept to use a medication adherence enhancing eHealth technology to enhance their AET. BC survivors are accepting to and willing to be supported during their AET, yet, the medication adherence enhancing eHealth technology needs to fit their needs and profiles. Thus, understanding acceptance profiles among BC survivors is fundamental for conceiving an effective medication adherence enhancing eHealth technology enhancing AET among BC survivors.

## Data Availability

The datasets presented in this article are not readily available because participants could be identifiable. The included tables provide the anonymized and summarized data. Requests to access the datasets should be directed to the corresponding author, catherine.goetzinger@gmail.com.

## Abbreviations

BC, Breast Cancer; AET, Adjuvant endocrine therapy.
