# Supplementary material for: Analysing breast cancer survivors’ acceptance profiles for using an electronic pillbox connected to a smartphone application using Seintinelles, a French community-based research tool
Source: Front Pharmacol. 2022 Sep 27;13:889695. doi: 10.3389/fphar.2022.889695 (PMC9551449; doi:10.3389/fphar.2022.889695)
Supplement: Supplementary file 1 [file DataSheet2.pdf]

Appendix 2 – e-survey inclusion criteria and e-consent

Inclusion criteria

- Women,
- who have (had) breast cancer for the first time in the last ten years,
- for which they received (at least temporarily) an oral hormone therapy (Tamoxifen®, Tamofen®, Nolvadex®, Femara®, Letrozole, Arimidex®, Anastrozole, Aromasine®, Exemestane, Fareston®, Kessar®...)

E-consent

Before you begin, please confirm that you meet the following inclusion criteria:
You are a woman: ☐ Yes /☐ No
You have been treated for breast cancer in the past 10 years: ☐Yes / ☐No
You have been treated at least temporarily with oral hormone therapy: ☐Yes /☐ No

If at least 1 No:
To ensure consistency in the responses collected, this survey is addressed exclusively to women who have had breast cancer in the past 10 years and have been treated with hormone therapy. We are therefore unable to continue the survey, thank you for your attention and understanding.

If yes:
Please confirm your consent to participate in the E-dherence survey:
☐Yes
☐No

The original version is in French, here is the English version translated with www.DeepL.com/Translator (free version)
