# Supplementary material for: Analysing breast cancer survivors’ acceptance profiles for using an electronic pillbox connected to a smartphone application using Seintinelles, a French community-based research tool
Source: Front Pharmacol. 2022 Sep 27;13:889695. doi: 10.3389/fphar.2022.889695 (PMC9551449; doi:10.3389/fphar.2022.889695)
Supplement: Supplementary file 2 [file DataSheet3.pdf]

Appendix 3 – Structure and definitions used in the e-survey

<u>Online questionnaire structure</u>

E-survey structure of sections	Variables asked within sections
I. Sociodemographic characteristics	• Age<br>• Marital status<br>• Children<br>• Educational level<br>• Professional status<br>• Financial situation
II. Health status and disease experience	• General health status<br>• Medication intake for other chronic diseases<br>• Quality of Life<br>• Year of diagnosis<br>• Disease impact on life<br>• Power to control disease<br>• Disease knowledge<br>• Disease recurrence

This work by Goetzinger et al. 2022 is licensed under a CC BY 4.0 license.

III. Medication adherence	• OHT adherence<br>• Motivation for OHT adherence/non-adherence<br>• Side effect management<br>• OHT management<br>• The implication of physicians in OHT adherence<br>• Satisfaction of information given by physician regarding<br>○ The nature of the treatment<br>○ Potential health benefits<br>○ Side-effects
IV. eHealth utilisation	• Possession of wearables/ health applications<br>• Utilisation frequency<br>• Reasons for use<br>• Important solutions for OHT management
Medication adherence enhancing eHealth technology	• Barrier to usability and acceptability<br>• Fascilitatiors of usability and acceptability

This work by Goetzinger et al. 2022 is licensed under a [CC BY 4.0 license](https://creativecommons.org/licenses/by/4.0/).
