# Supplementary material for: Analysing breast cancer survivors’ acceptance profiles for using an electronic pillbox connected to a smartphone application using Seintinelles, a French community-based research tool
Source: Front Pharmacol. 2022 Sep 27;13:889695. doi: 10.3389/fphar.2022.889695 (PMC9551449; doi:10.3389/fphar.2022.889695)
Supplement: Supplementary file 3 [file DataSheet1.pdf]

Appendix 1 – Participant information sheet of the E-dherence survey

Madam,

The Edherence survey was developed by Prof. Huiart and Prof. Mancini. The study is conducted at Seintinelles under the responsibility of the Luxembourg Institute of Health.

This note is a written document to help you decide whether or not to participate in this study.

You are free to decide whether or not to participate in this complimentary study, regardless of your medical follow-up.

You can take time to think about your participation in this research, and discuss it with your doctor and your family.

Pr Laetitia HUIART, principal investigator (E-mail : laetitia.huiart@lih.lu) and Pr Julien MANCINI (julien.mancini@univ-amu.fr) are at your disposal to answer all your questions and to explain what you do not understand.

1. INFORMATION

The purpose of this document is to provide you with the written information necessary for your decision. We thank you for reading it carefully.

Purpose of the research: We would like to get your input on what could be done to support women who are on oral hormonal therapy (often-called hormone therapy) after breast cancer. We are particularly interested in your opinion on e-health applications.

Therefore, we invite you to take part in this survey:

→ Women,

→who have (had) breast cancer for the first time in the last ten years,

→for which they received (at least temporarily) an oral hormone therapy (Tamoxifen®, Tamofen®, Nolvadex®, Femara®, Letrozole, Arimidex®, Anastrozole, Aromasine®, Exemestane, Fareston®, Kessar®...)

For those who have had a recurrence or second cancer, please answer the questions based on your experience with your first breast cancer diagnosis.

2. CONDUCT OF THE RESEARCH

This is an online questionnaire survey. The questionnaire consists of about 30 questions and will require your attention for at least 20 minutes.

The questions will focus on characteristic elements of your personal situation, in order to know you better, as well as on your experience of the disease, of hormone therapy and finally, on your opinion concerning the use of new technologies to accompany patients in their medical follow-up.

If you were to interrupt the questionnaire, you can save your answers and continue later.

3. CONFIDENTIALITY AND DATA PROCESSING

The results of all participations to this questionnaire will be grouped together to be analyzed and all your answers will remain strictly anonymous. We, therefore, guarantee the confidentiality of the answers you provide.

These data will be kept for 5 years to allow their full exploitation.

The study will be conducted in accordance with the European and French laws in force concerning research involving the human person and the protection of personal data, in particular, Regulation (EU) 2016/679 of the European Parliament and of the Council of 27 April 2016 and the Data Protection Act No. 78-17 of 6 January 1978 as amended by Act No. 2018-1125 of 12 December 2018 on the protection of individuals with regard to the processing of data (RGPD)

At no time will your collected data be transmitted outside the European Union.

For more information, contact the data protection officers at the Seintinelles coordination center or the Luxembourg Institute of Health.
In accordance with articles L. 1121-1 and following the Public Health Code, this project has been studied by the Comité de Protection des Personnes Sud-Est III, which issued a favourable opinion.
The results of this research may be presented at conferences or in scientific publications. However, your personal data will not be identifiable in any way because no identifying data will be kept.
In application of the provisions of article L 1111-7 of the Public Health Code, you may, if you wish, be kept informed of the overall results of this research, by contacting directly Pr Laetitia Huiart (e-mail: laetitia.huiart@lih.lu) or Pr Julien Mancini (julien.mancini@univ-amu.fr). The people in charge of the research will then be able to explain to you the main results of the survey as well as the impact of the study.

If you are not satisfied despite the commitment to respect your rights and protect your data, you can lodge a complaint with the supervisory authority: the Commission Nationale de l'Informatique et des Libertés (https://www.cnil.fr/fr/cnil-direct/question/adresser-une-reclamation-plainte-la-cnil-quelles-conditions-et-comment).

4. ACCESS TO PERSONAL DATA
The French National Commission for Information Technology and Civil Liberties (CNIL) provides for the right to "data portability", which means that you have the right to access (for your personal use) your data collected and computerized in the framework of the survey. You also have the right to make or request a transfer of your personal data from one organization to another.

5. CONSENT
Your participation is voluntary: you are free to accept or refuse to participate in this research.
If you decide to participate, you may withdraw your consent to the research at any time without liability or prejudice. We will simply ask you to inform the person in charge of the research. You will not have to justify your decision.
For any questions concerning these rights, you can contact the researchers involved in this research.
Thank you in advance for your participation.

The research team of the study

The original document is in French, here is the English version translated with www.DeepL.com/Translator (free version)
